# Asymmetric distribution of biomolecules of maternal origin in the *Xenopus laevis* egg and their impact on the developmental plan

**DOI:** 10.1038/s41598-018-26592-1

**Published:** 2018-05-29

**Authors:** Radek Sindelka, Pavel Abaffy, Yanyan Qu, Silvie Tomankova, Monika Sidova, Ravindra Naraine, Michal Kolar, Elizabeth Peuchen, Liangliang Sun, Norman Dovichi, Mikael Kubista

**Affiliations:** 1 Institute of Biotechnology of the Czech Academy of Sciences - BIOCEV, Prumyslova 595, Vestec, 252 50 Czech Republic; 2 Department of Chemistry and Biochemistry, University of Notre Dame, Notre Dame, IN 46556 USA; 3 Institute of Molecular Genetics, Videnska 1083, 142 20 Prague 4, Czech Republic; 4 TATAA Biocenter, Odinsgatan 28, Göteborg, 411 03 Sweden; 5 Present Address: Department of Chemistry, Michigan State University, East Lansing, MI 48824 USA

## Abstract

Asymmetric cell division is a ubiquitous feature during the development of higher organisms. Asymmetry is achieved by differential localization or activities of biological molecules such as proteins, and coding and non-coding RNAs. Here, we present subcellular transcriptomic and proteomic analyses along the animal-vegetal axis of *Xenopus laevis* eggs. More than 98% of the maternal mRNAs could be categorized into four localization profile groups: animal, vegetal, extremely vegetal, and a newly described group of mRNAs that we call extremely animal, which are mRNAs enriched in the animal cortex region. 3′UTRs of localized mRNAs were analyzed for localization motifs. Several putative motifs were discovered for vegetal and extremely vegetal mRNAs, while no distinct conserved motifs for the extremely animal mRNAs were identified, suggesting different localization mechanisms. Asymmetric profiles were also found for proteins, with correlation to those of corresponding mRNAs. Based on unexpected observation of the profiles of the homoeologous genes *exd2* we propose a possible mechanism of genetic evolution.

## Introduction

One of the most fascinating and puzzling questions in biology is how complex organisms consisting of tissues composed of numerous cell types develop after the fusion of just two cells – the sperm and the egg. Eggs, especially those originating from mammals, have traditionally been described as rather unsophisticated cells in terms of biomolecule localization. However, in recent years, oocytes have been found to have complex internal structures with precise cellular polarity features that are essential for successful fertilization and fetal development.

The most popular animal models for the study of asymmetry governing early development aside from lower organism such as *Drosophila*^1–3^ are oocytes and early stage embryos from amphibians and fish. The first experiments with amphibian oocytes and eggs were published in the late 1980s^4–6^. For mammalian oocytes and eggs, distinguishable outer features that would reflect asymmetric distribution of cell fate determinants have so far not been found, possibly due to their small size, which makes localization studies challenging. Arguably the most popular model to study oocyte and egg asymmetry is the African clawed frog, *Xenopus laevis*. Its eggs are large (~1.3 mm), harbor very high concentration of RNA (~4 μg), and contain copious amounts of proteins (~130 µg), of which ~13 µg are proteins other than yolk proteins^7^.

In *Xenopus laevis*, the first developmental axis is formed during oogenesis and is referred to as the animal-vegetal axis. Both its poles show distinct features: the animal hemisphere is dark due to the presence of melanin pigment granules and contains the nucleus, while the vegetal hemisphere is light and consists mainly of yolk proteins. Cell fate determinants distributed asymmetrically along the animal-vegetal (A-V) axis are translated during the early steps of development, which leads to the formation of the germ layers^8^. These layers consist of ectodermal cells that derive from the animal part, mesodermal cells from the equatorial region, and endodermal cells from the vegetal part of the egg.

The localization of maternal mRNAs into the vegetal hemisphere has been studied by many laboratories, and two distinct pathways have been described. One group of transcripts actively localizes during the early phases of oogenesis by the METRO (messenger transport organizer) pathway and includes germ plasm determinants such as *nanos1* (also known as *xcat2*), *dazl*, *ddx25*, *pgat* (also called *xpat*), and *wnt11*^9^. Genes such as *trim36*, *rras2*, *sybu*, and *germes* were found localized to the vegetal cortex, but their roles during early development are not completely understood. The second group is localized by the late transport pathway that creates a shallow vegetal gradient for transcripts such as *gdf1* (also called *vg1*) and *vegt*, which code primarily for a member of the TGF family and a transcription factor, respectively. These genes are important for germ layer specification and patterning. Recently, some vegetal candidates including *rbpms* (also called *hermes*) and *plin2* (also called *fatvg*) were found to localize by both the early and late pathways^8^. The first localization element identified was a 340-nucleotide fragment within the 3′UTR of *gdf1* mRNA^10^. Other studies analyzed the localization elements in *nanos1* 3′UTR^11^. These contain repeats of conserved short sequences, referred to as localization motifs, called VM1 ([UC][UC]UCU) and E2 ([UA][UC]CAC) that were predicted to bind RNA-binding proteins required for active transport^12–15^. Subsequent functional experiments revealed that the localization motifs alone are insufficient to regulate transportation; surrounding sequences are also essential^15^.

Recently, several transcripts were found to be enriched in the animal part of the oocyte and were suggested to be actively localized, though by an unknown mechanism (reviewed in^16^). We performed detailed analysis of the spatial distribution of these mRNAs using qPCR tomography and proposed that they are produced in the oocyte germinal vesicle, which is located in the animal hemisphere, and diffuse during oogenesis, without active transport, producing the observed accumulation in the first 1/3 of the egg from the animal pole^17,18^.

Fertilization of the *Xenopus* egg leads to an internal structural rearrangement and initiates cell division. *De novo* transcription, however, remains paused for most of the genes until the mid-blastula transition (MBT), even though partial zygotic gene expression starts earlier^19^. An interesting feature of *X. laevis* compared to other model organisms is its early specification of the second and third developmental axes. The first cell division separates the embryo into the left and right blastomeres that propagate into the left and right (L-R) halves of the embryonic body. The second cell division determines the dorsal and ventral parts of the embryo (D-V). The specification of the L-R and D-V developmental axes does not seem to be induced by mRNA asymmetry^20,21^. Instead, other types of molecules are expected to be involved. Recent studies revealed a broad spectrum of cell fate determinants, including small non-coding RNAs, proteins, and metabolites^18,22–24^.

The introduction of high-throughput profiling methods such as microarrays^25^, RNA-sequencing^26^, deep quantitative proteomics^7,27^, and metabolomics^24^ in studies of *Xenopus* has made global profiling possible. Improved methods for sample preservation, precise cryo-sectioning, and efficient isolation of biomolecules have made high-resolution spatio-temporal analysis of embryonic development possible and has been used to study *Xenopus* oocytes^28^ and zebrafish embryos^29^.

Here we perform RNA and protein localization studies using a combination of modified Tomo-Seq and deep quantitative proteomic analysis to uncover the localization profiles of the main types of RNAs and proteins within *X. laevis* eggs and in stage 8 blastula embryos, which are entering the mid-blastula transition (MBT) and have a distinct A-V axis. Analysis of intracellular RNA and protein profiles revealed novel conserved 3′UTR motifs that are putative localization motifs.

## Results

### Classification of intracellular gradients

RNAs were divided into four localization categories: extremely animal, animal, vegetal and extremely vegetal, based on the criteria in Table 1. Proteins were divided into only three localization categories that is animal, vegetal and even, since the current resolution did not reveal any maternal proteins with extreme profiles.

**Table 1:** Criteria for the classification of RNAs into localization categories based on intracellular profiles.

Localization Category	Criteria
Extremely animal RNAs	maximum in A; (A + B) > (D + E); C > D or E
Animal RNAs	maximum in B; D + E < 40% of all transcripts
Vegetal RNAs	(D + E) > (A + B + C); D > A or B or C
Extremely vegetal RNAs	E > 50% of all transcripts; E > 2*D
Other RNAs	do not meet any of the selection criteria above

### Intracellular distributions of mRNAs

**Figure 1:**
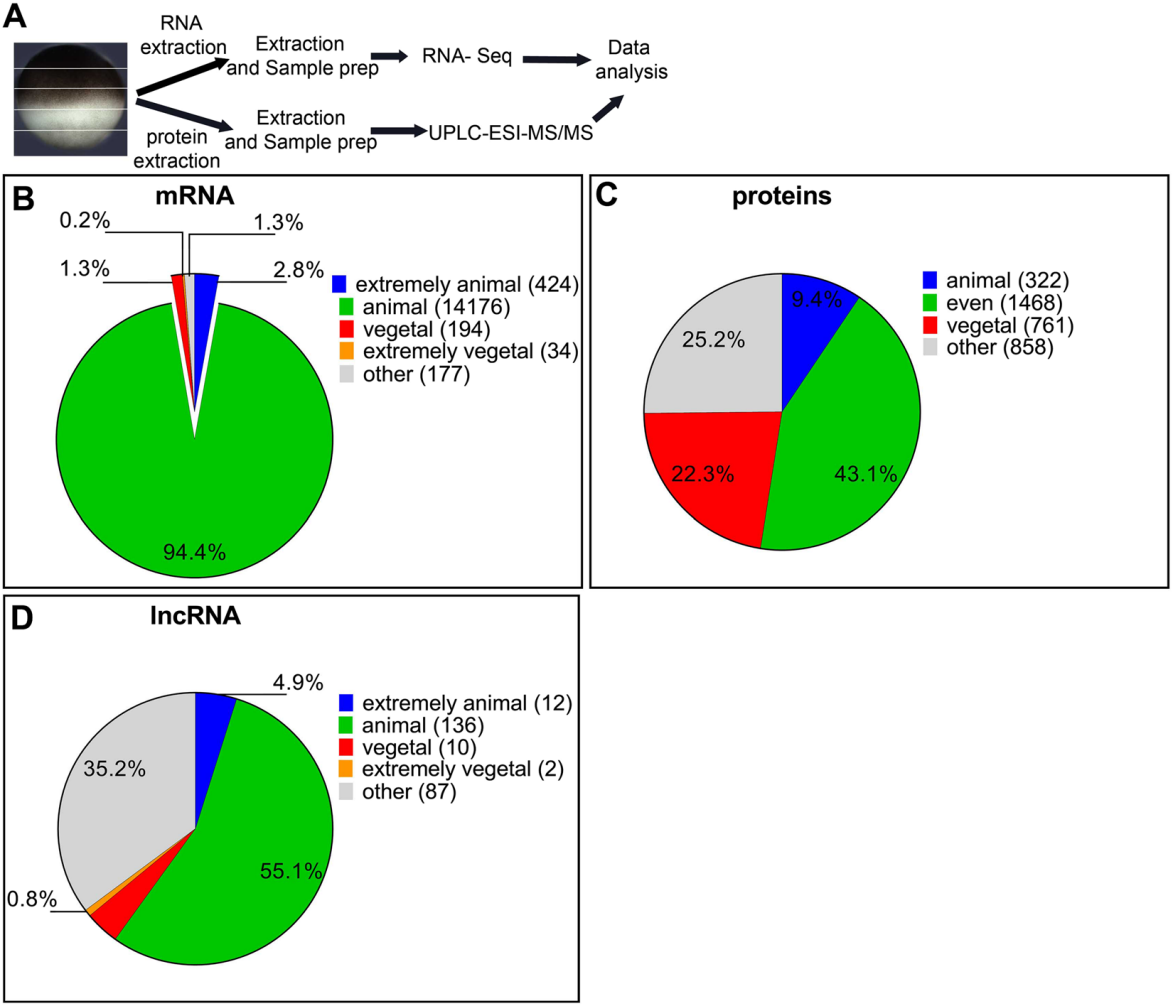
Classification of the studied biomolecules into localization categories based on their intracellular profiles. Experimental scheme (**A**). Classification of 15005 mRNAs (**B**), classification of 3409 proteins (**C**) and classification of 247 long noncoding RNAs (**D**).

**Figure 2:**
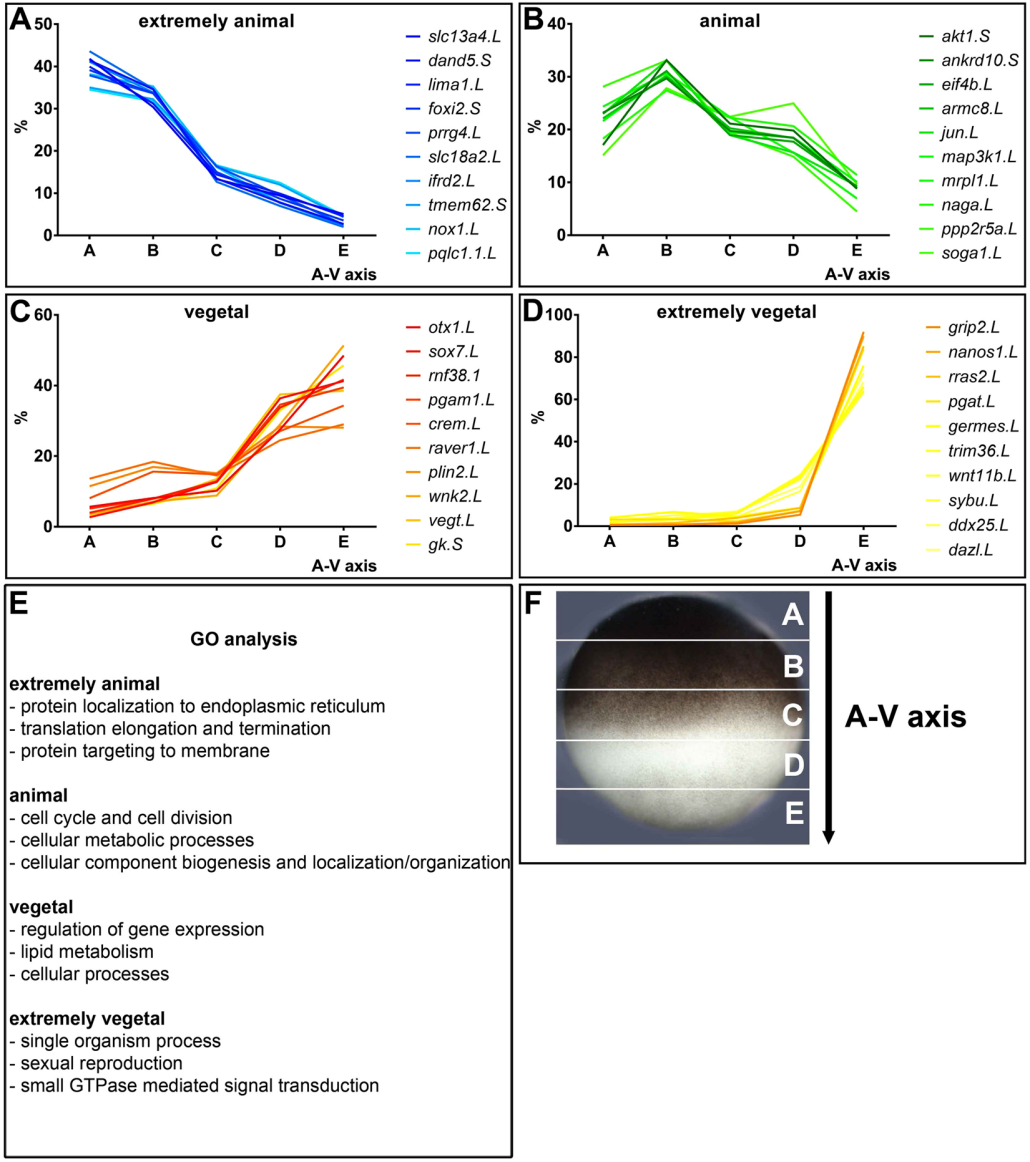
Intracellular profiles for selected extremely animal (**A**), animal (**B**), vegetal (**C**) and extremely vegetal (**D**) mRNAs. Predicted functions based on gene ontology (GO) analysis (**E**). Scheme of A-V sectioning (**F**).

15005 mRNAs were identified from the RNA-Seq data. All the 15005 mRNAs, including those with extreme polarizations, were present in at least some copies in all of the segments along the animal-vegetal axis (Fig. 1B). The extremely animal mRNAs show a gradually increasing concentration gradient, with a maximum at the animal pole. The animal mRNAs accumulate in the first third of the egg from the animal pole at the expected location of the nuclei. The vegetal mRNAs show a gradual increase from the animal to the vegetal pole and the extremely vegetal mRNAs accumulate at the vegetal cortex and are most abundant in the last vegetal segment (Fig. 1B, Supplement File 1,^17^). The vast majority of the mRNAs (94.4%) show animal localization and were of all kinds. 2.8% of the mRNAs have an extremely animal polarization and the majority of these code for translation and transcription regulators. Some are also known to control protein localization. Vegetal mRNAs represent 1.3% of the total and most of them code for transcription regulators involved in germ layer specification and proteins involved in lipid metabolism. The latter have previously been associated with the METRO pathway^16^. Extremely vegetal mRNAs make up 0.2% of the total and include transcripts with functions during germ plasm determination, sexual reproduction, and the regulation of the development of the organism (Fig. 2).

### Intracellular distributions of proteins

Deep proteome analysis identified more than 3409 maternal proteins in the *X. laevis* eggs. 9.4% were classified as animal, 22.3% were vegetal, 43.1% showed even localization. Even though our criteria were defined to determine maximum protein profiles with clear trends, low resolution and reproducibility of proteome analysis resulted in the remaining 25.2% of the proteins referred to as having “other profile” (Fig. 1C).

### Intracellular distributions of lncRNAs

LncRNAs were categorized using the same criteria as for the mRNAs. In total, 247 lncRNAs were identified in the mature *X. laevis* eggs based on lncRNA prediction from *de novo* transcriptome analysis. 4.9% of those showed the extremely animal, 55.1% the animal, 4.0% the vegetal, and less than 0.8% showed the extremely vegetal profile (Fig. 1D).

### mRNA gradients in the blastula stage

**Figure 3:**
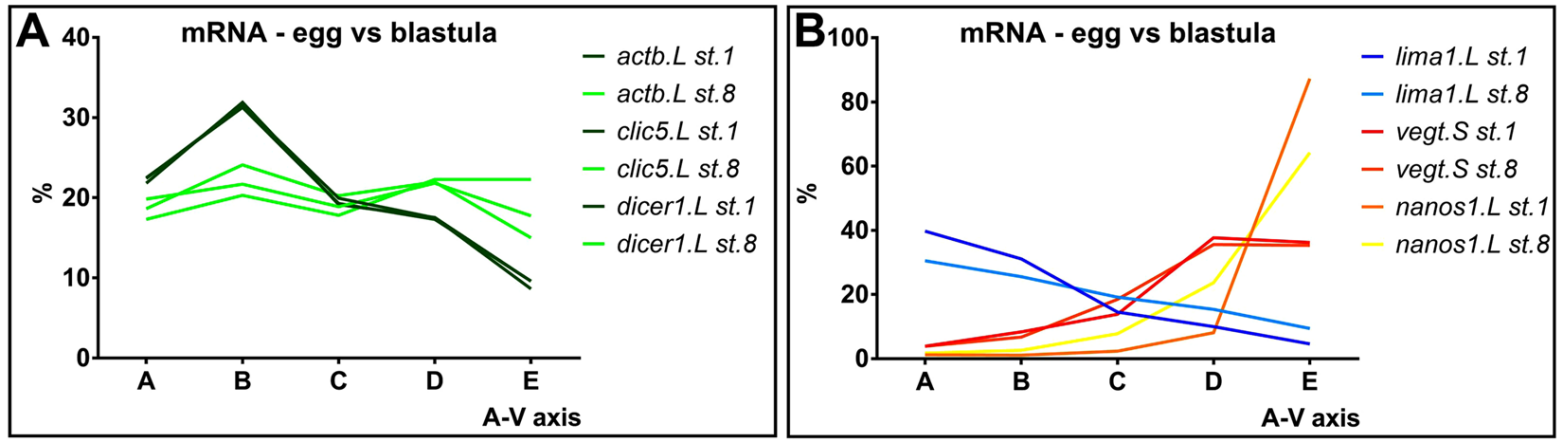
Animal mRNAs become evenly distributed along the A-V axis at blastula stage (**A**), while extremely animal, vegetal, and extremely vegetal mRNAs remain asymmetrically distributed at blastula stage (**B**).

Using qPCR tomography, we found that the localization profiles for genes with animal polarization, represented by *actb*, *clic5* and *dicer1*, in the egg become uniform across the embryo at blastula stage (Fig. 3A). This contrasts the profiles of actively transported genes, such as the extreme animal and vegetal RNAs, that remain asymmetrically distributed across the embryo in later stages (Fig. 3B).

### Localization motifs

**Figure 4:**
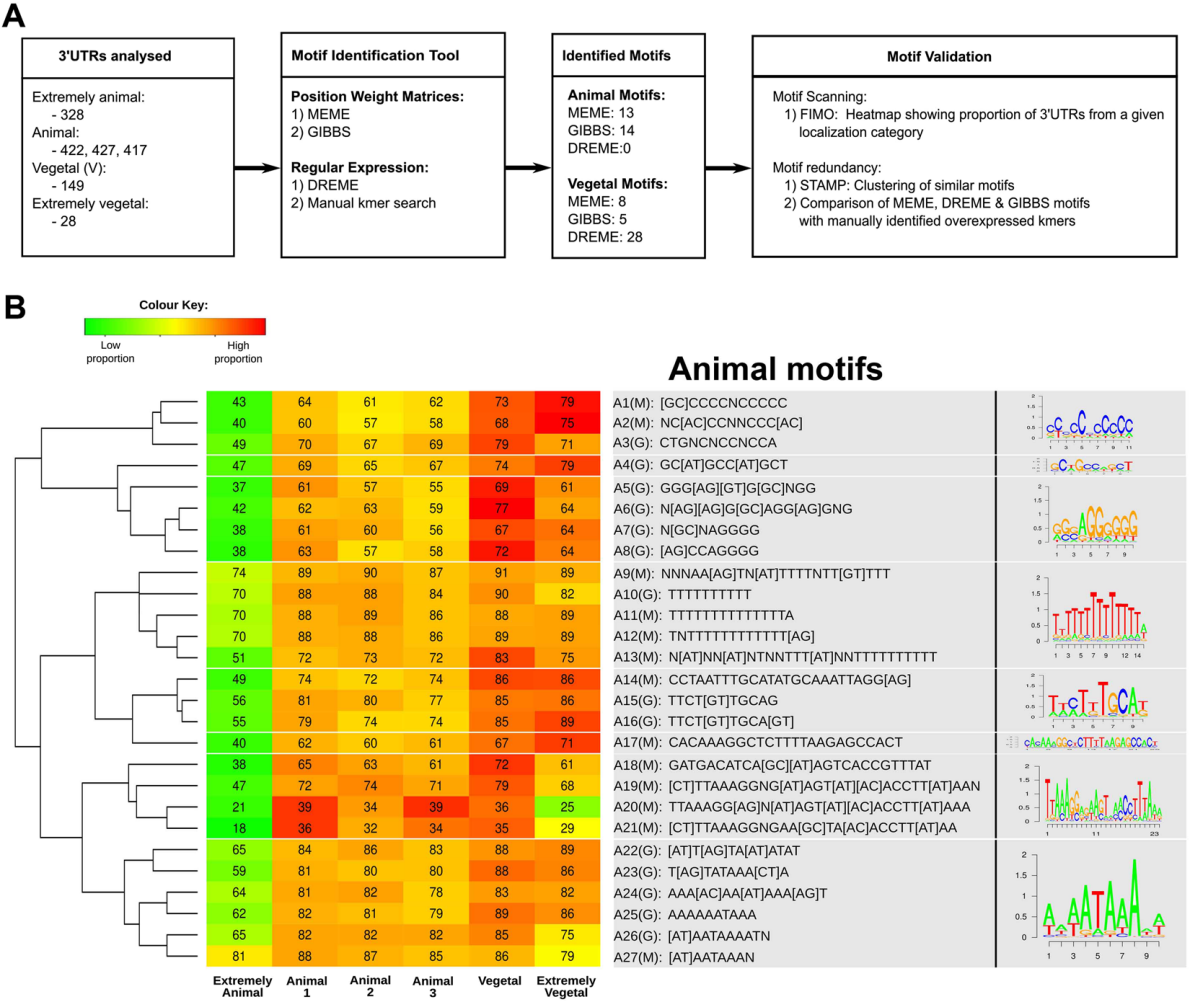
Scheme for the identification of localization motifs (**A**). Cluster analysis of the animal localization motifs identified using MEME (A1M-A27M) and GIBBS (A3G-A26G). Heatmap indicates the proportion of motifs and the numbers the percentage of the 3′UTRs that contains the motifs (**B**).

**Figure 5:**
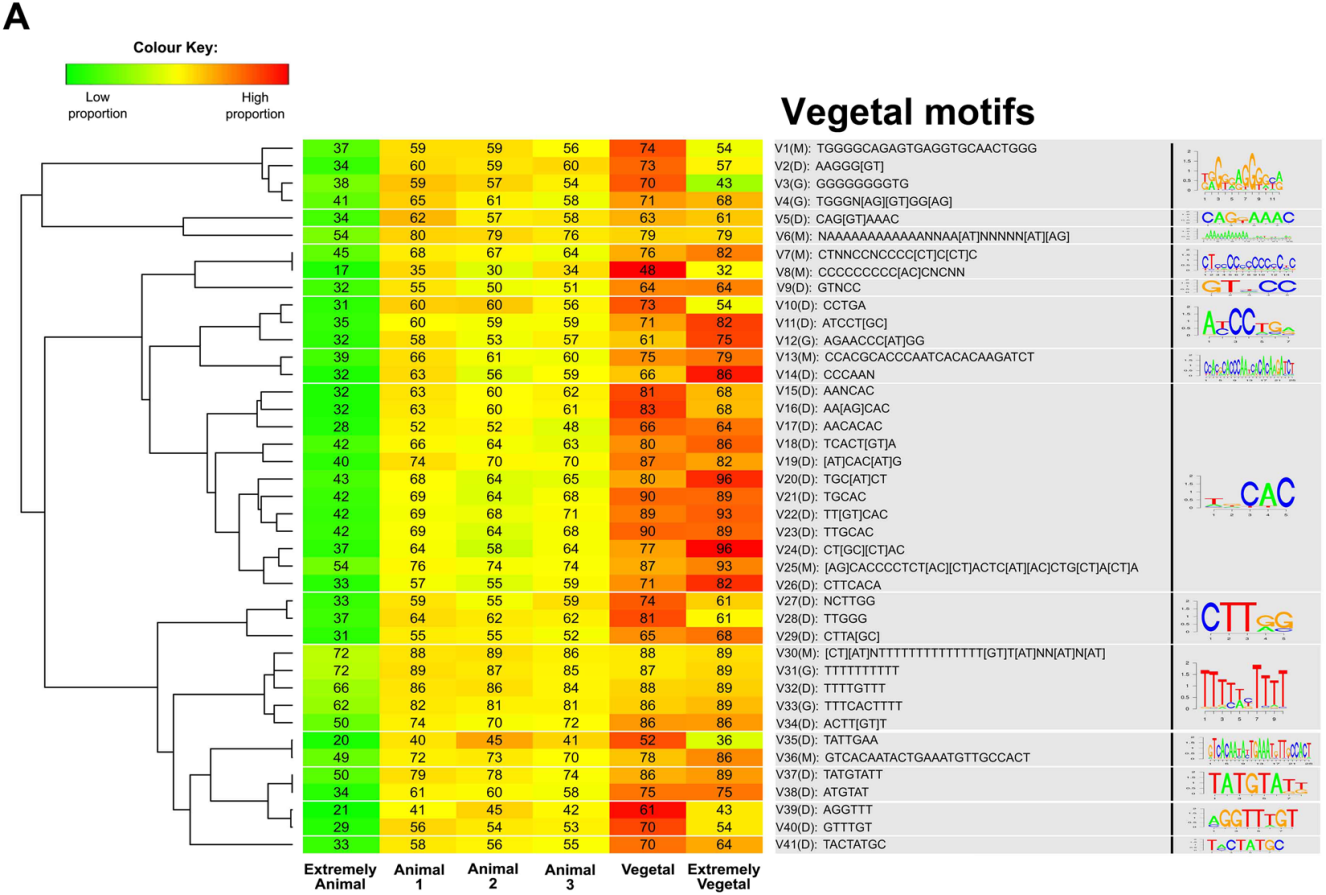
Cluster analysis and motif validation for the motifs behind vegetal localization identified using MEME (V1M-V36M), GIBBS (V3G-V33G) and DREME (V2D-V41D). Heatmap indicates proportion of motifs and numbers reflect percentage of 3′UTRs containing at least one copy of a motif (**B**).

3′UTRs among the extremely animal, vegetal, and extremely vegetal mRNAs were analyzed for consensus sequences that could serve as localization motifs using the analysis packages MEME, GIBBS, and DREME. In total, 27 tentative animal motifs for extremely animal mRNA localization, 41 tentative vegetal motifs for vegetal, and 41 for extremely vegetal localizations were found (Figs 4 and 5, respectively). Cluster analysis to identify related motifs and to reduce the number of variants into families was performed and resulted in 8 families of motifs for the extremely animal mRNAs and 14 families for the vegetal and extremely vegetal mRNAs.

### Animal localization motifs

Among the animal motifs, three families contain uniform sequences of either long poly-C (A1-3), poly-U (A9-13), or G-rich motifs (A5-8). Four families contain long and rather variable sequences: GC[AU]GCC[AU]GCU (A4), UUCU[GU]UGCA (A14-16), CACAAGGCUCUUUUAA (A17) and UUAAAGG (A18-21), and the last family contains a highly conserved AAUAAA sequence (A22-A27). FIMO analysis compared the number of genes per localization category that contains at least one copy of the motifs and found that none of the animal motifs were enriched in the extremely animal localization category.

### Vegetal localization motifs

Vegetal motifs were clustered based on sequence conservation into 14 families. Four families contain either long uniform sequences G-rich (V1-4), poly-A (V6), poly-C (V7-8) or poly-U (V30-34). Ten families have short conserved sequences: CAG[GU]AAAC (V5), GUNCC (V9), A[UC]CC[AU] (V10-12), CCCAA (V13-14), CAC-rich (V15-26), CUU[GA][GC] (V27-29), UA[CU]UGAA (V35-36), UAUGUA (V37-38), AGGUUU (V39-40) and UACUAUGC (V41). FIMO analysis revealed significant enrichment of most of the motifs in the 3′UTRs of the vegetal and extremely vegetal mRNAs.

### Kmer analysis

Kmers of three to seven nucleotides in length were analyzed for overrepresentation within the 3′UTRs of extremely animal, vegetal, and extremely vegetal mRNAs (Table 2). 54 and 62 3-mers were overrepresented (p = 0.001) among the animal and vegetal sequences, respectively. However, even though these 3-mers were overrepresented, their presence was not exclusive to any one localization category, rather they were found in several of the localization categories.

**Table 2:** Number of significantly overexpressed and unique kmers in the 3′UTR of genes from the animal and vegetal regions.

Kmer length	Significant Animal kmers			Significant Vegetal kmers		
	Over-represented	Unique to animal	Unique kmers found within the identified (MEME, DREME, GIBBS) motifs	Over-represented	Unique to vegetal	Unique kmers found within the identified (MEME, DREME, GIBBS) motifs
3-mer	54	0		62	0	
4-mer	154	1	ACCA	224	9	CTTA, GTTA, TAGA, ATAG, ACCT, GGCA, GTGC, TGGC, CTCA
5-mer	343	7	GATAA, TGGTG, GACTG, GACAA, TGTCC, TCATC	677	72	CCACT, TACTC, ATCAC, GCACC, TCCAC, TCACC, ACCCA, GCTCT, CCACA, CACCT, AGCCA, ACCTG, GCCAC, ACTCC, TTACC, GATCT, GATCA, GGGGA, TGATC, TAAGG, TACCC, GTCTC, GAGTC, GTCCC, TGGGT, CAACT, GGGTT, TGAAC, GTATG, GCCAT, GGTTA, GGGCA, ACAGC, GGCAG, ACAAC, TAGGG, CAGCC, GCTTC, ATCCC, GCAAC, ATGAC, AGATC, AGCCT, AGGTG, GGGTG, GGTAT, GTGAG, GGGCT, CATCC, GCTAA, GGTGC, GGGTA, TGAGG, GTTAG, GAGTG, GAGGT, CTATC, GATAG, GCCTC, CTAGG, GCTAG, CCGAA, CGATT, TCGGC, CGTAT, CACTA, CTCAC, ACTCA, GTCAC, CACTC, TCTCA, AGAGT
6-mer	1327	104		1458	196	
7-mer	5584	971		5263	717	

Nearly twice the number of 4-mers were overrepresented among vegetal compared to animal 3′UTRs (224 vs 154), out of which nine vegetal 4-mers were found only within the vegetal 3′UTRs, while one 4-mer was exclusive to the animal 3′UTRs. These are referred to as unique kmers for the particular localization category. Unique 4-mers overlap with the motif sequences identified from DREME, MEME, and GIBBS analyses. The largest difference between animal and vegetal kmer overrepresentation and uniqueness was among 5-mers. 343 5-mers were overrepresented in the animal 3′UTR, while 677 were overrepresented in the vegetal 3′UTRs. Only seven of the animal 5-mers and 72 of the vegetal 5-mers were unique to the animal and vegetal 3′UTRs, respectively. Comparison with the motifs identified by DREME, MEME and GIBBS (Figs 4 and 5) found six out of the seven animal 5-mers and all of the 72 vegetal 5-mers. There was minimal difference in overrepresentation or uniqueness among 6- and 7-mers.

### Detailed analysis of motif location in 3′UTRs and frequency dependence on motif length

The positions of a few selected vegetal motifs were extracted from the FIMO analysis and plotted onto the relevant 3′UTR sequences to compare the motifs between the three extremely animal (*lima1*, *dand5*, and *ifrd2*) and the three extremely vegetal mRNAs (*ddx25*, *grip2*, and *nanos1*) with the steepest localization profiles (SFig. 3A). Selection of representative genes was based on either the similarity of their UTR lengths to that of the mean UTR length of their given profile or the fact that they represented well characterized genes with previously described localization profiles. Overall, the extremely vegetal mRNAs show higher prevalence of localization motifs than the extremely animal mRNAs, suggesting that the 3′-UTR sequence features may overall be more important for vegetal localization, but the results are gene dependent implying other factors are contributing to the polarization.We also tested the importance of the lengths of the motifs, comparing the frequencies of the vegetal motifs CAC-rich and A[UC]CC[AU]. In this comparison, we found no difference in enrichment between the given (5-mer) motif and longer forms.

### Homoeologous mRNAs

**Figure 6:**
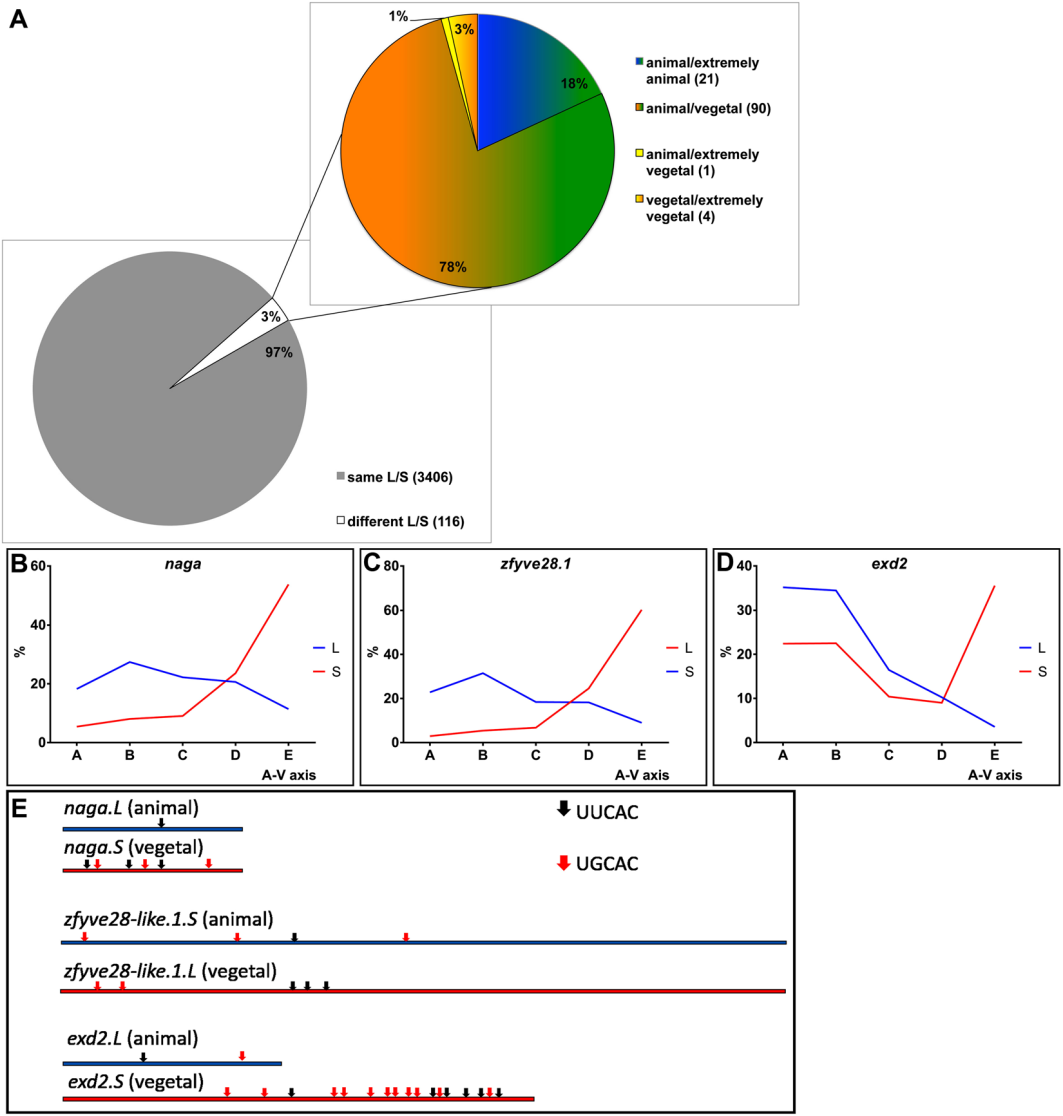
Analysis of homoeologous genes that show contrasting polarization. Out of more than 3500 homoeologous maternally expressed genes less than 100 showed different localization of its L and S versions. (**A**) Localization profiles of the L and S versions of *naga*, *zfyve28* and *exd2* (**B**–**D**). Presence of vegetal localization motifs UUCAC and UGCAC in the 3′UTRs of *naga*, *zfyve28* and *exd2* (**E**).

*Xenopus laevis* is an allotetraploid with more than 56.0% of its coding genes existing as homoeologues^30^. The maintained homoeologous chromosomes, which represent the remnants of the two ancestral subgenomes, can be distinguished easily by their differing lengths and are referred to as either L (long chromosomes) or S (short chromosomes). The L and S forms of the homoeologous genes can usually be annotated based on single nucleotide polymorphism present in their coding sequence^30^. The high read depth of our Tomo-Seq methodology allowed us to discriminate between the two variants. In total, 3500 pairs of homoeologous maternal mRNAs were identified in the *Xenopus* egg. 97.0% of these shared the same localization profile (Fig. 6A), indicating that the localization motif/mechanism for these homoeologues are still conserved. The remaining 3.0% showed contrasting localization profiles, with only a few of these showing diametrically opposite profiles, for example one being vegetal/extremely vegetal while the other is animal/extremely animal (Supplement Table 2).

Three homoeologue pairs that showed contrasting localization profiles: *naga*.(*L/S* -Fig. 6B), *zfyve28-like1*.(*L/S* - Fig. 6C), and *exd2*.(*L/S* - Fig. 6D) were selected for more detailed sequence analysis (Fig. 6E). For each of those pairs we found a much higher variation in the 3′UTR than in the coding region. Analysis of the presence and amount of the vegetal localization motifs UUCAC and UGCAC within the 3′UTR of these genes, showed that the UUCAC motif is present in at least 3-fold higher abundance in the vegetal localized version of the gene compared to the animal localized version. This is also observed for the UGCAC motif, where an even higher enrichment was observed in the 3′UTR of the vegetal localized version of the genes *naga* (3:0) and *exd2* (11:1) but slightly opposite for *zfyve28-like1* (2:3).

### Protein distribution

**Figure 7:**
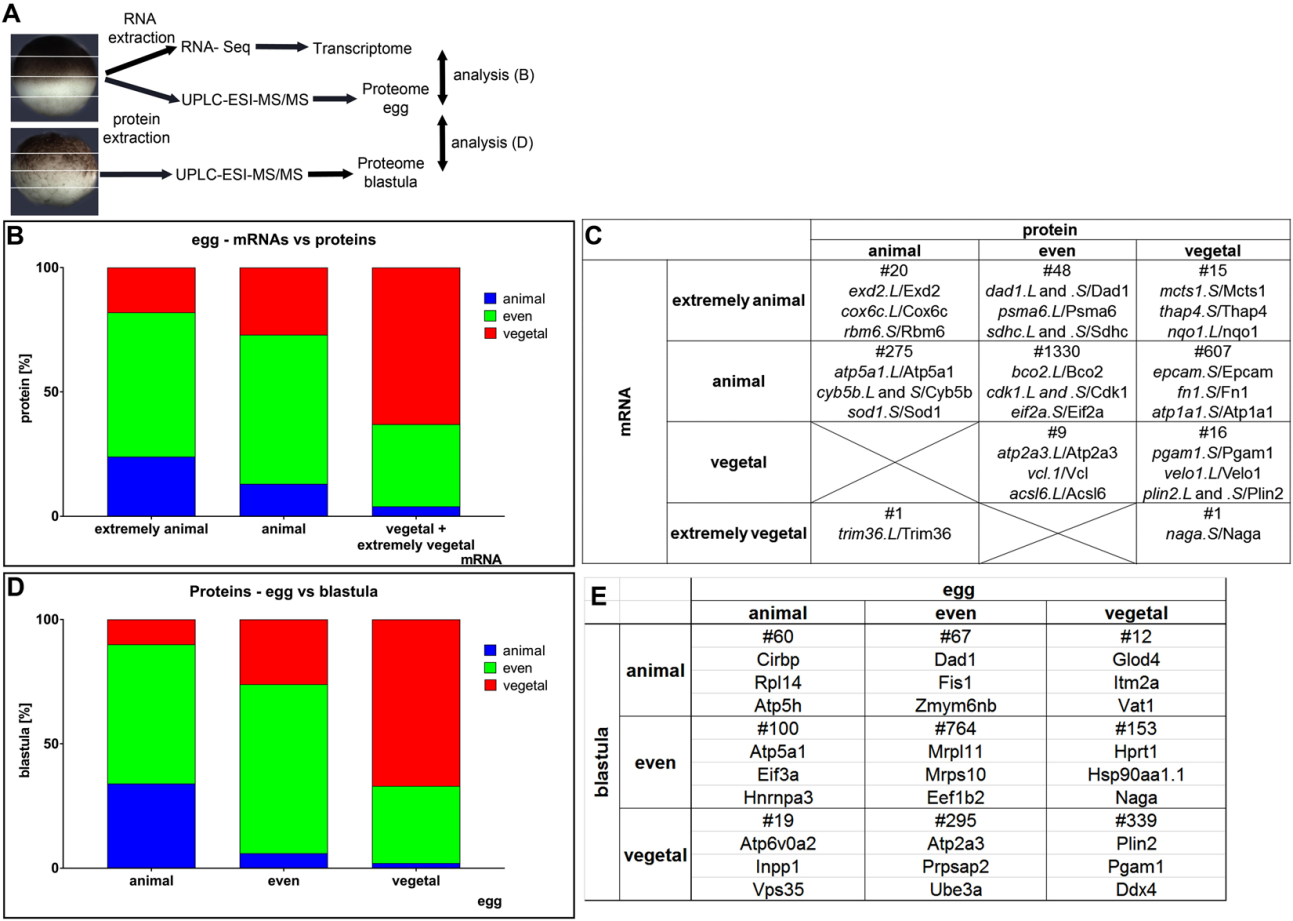
Proteome profiling along the A-V axis of *Xenopus laevis* eggs. Schematic showing the experimental design (**A**). Protein localization and comparison with mRNA (**B**). Selected candidates with similar/different localization at mRNA/protein levels with known interesting biological functions (**C**). Comparison of protein localization at egg and blastula (**D**). Examples of egg and blastula specific proteins (**E**).

Deep proteome quantitative profiling was performed along the animal-vegetal axis of the *Xenopus* egg divided into four segments, Fig. 7A, as described in Materials and Methods. Resolution, sensitivity and dynamic range were sufficient to categorize the proteins as animal, vegetal, or even, but we could not positively identify extreme polarizations. The proteins were categorized based on the measured profiles as: (1) animal, characterized by most of the protein (>50%) being localized to segments A and B; (2) even, protein amounts show minimal variation across segments; (3) vegetal, characterized by most of the protein being present in segments C and D. Proteins that did not meet any of these criteria were classified as having “other” profile.

Two developmental stages: egg and blastula, each measured in biological triplicates, were compared. The protein localization profiles for the egg are shown in Fig. 1C. In total 3409 proteins were identified in at least two replicates of the egg samples, and 3145 proteins in at least two replicates of the blastula samples. The egg and blastula stages shared 2827 proteins (Supplement Table 3). 9.4% of the proteins in the egg showed animal, 43.1% showed even, and 22.3% showed vegetal profiles. Proteins in the blastula stage showed in 6.3% animal, in 39% even, and in 27.2% vegetal profiles.

Egg protein localization profiles were compared to the profiles of the corresponding mRNAs (Fig. 7B). For the egg samples: 24.1% of the proteins coded by extremely animal mRNAs displayed an animal protein profile, 57.8% even protein profile, and 18.1% a vegetal protein profile. Proteins derived from animal mRNAs were mainly evenly localized (60.2%), with smaller numbers classified as vegetal (27.4%) or animal (12.4%). Proteins coded for by vegetal mRNAs most often showed a vegetal profile (63.0%), some showed even (33.3%), and only 3.7% showed animal polarization. Three genes were selected from each category for illustration of the measured data (Fig. 7C). Interestingly, for about 10% of the identified proteins (223), the corresponding mRNAs were not present in the Tomo-Seq data (Supplement Table 4). 12.0% of the proteins with undetected mRNAs were animally localized, and 53.0% of the proteins without corresponding mRNAs were vegetal and included parts of complement, apolipoproteins, and other developmentally important genes. These data suggest that these proteins were produced already during oocyte maturation and do not need to be replenished. The mRNAs coding for these proteins have been degraded.

Deep proteome spatial analysis was performed also on blastula embryos (stage 8) and compared to the egg data (Fig. 7D). 33.5% of the egg animal proteins remain localized animally, while 55.9% have even distribution. Only 10.6% of the proteins produced a vegetal profile. Proteins showing an even profile in the egg remain even in 67.9% of cases, become vegetal in 26.1% of cases and were animal in 6.0 % of cases at the blastula stage. Vegetal proteins in the egg remain vegetal in 67.2% of the cases in the blastula stage, are localized animally in 2.4% and evenly in 30.4% of the cases. Animal profiles were shared for several ribosomal proteins, even profiles were shared for the chaperonin complex components, mitochondrial ribosomal proteins, heat shock proteins, proteasome subunits, some ribosomal proteins, and elongation factors, and vegetal profiles were shared for several important proteins including Plin2 and members of Cathepsin family. 582 of the proteins found in the egg were not seen in the blastula stage and include, for example, maternal Velo1, while 318 proteins were unique for the blastula samples and include, for example, animal Sumo3 and vegetal Zfyve28-like (Supplement Table 5). Three selected proteins from each category are presented in more detail in Fig. 7E.

## Discussion

Biomolecules such as non-coding RNAs, mRNA, and proteins have important functions as cell fate determinants during embryogenesis. Here we have performed the first exhaustive study of the global mRNA, lncRNA and protein intracellular profiles within the *Xenopus laevis* egg and measured profiles in the blastula stage.

In a previous study using RT-qPCR, we identified three distinct intracellular profiles: animal, vegetal and extremely vegetal, for a small set of mRNAs^28^. The global profiling performed here using RNA-Seq and UPLC-ESI-MS/MS suggests these profiles are universal for at least mRNAs and lncRNAs, and probably also for proteins, where uncertainty is higher due to lower resolution. In contrast to the historical view of a rather homogenous egg with minor active localization consisting of a few hundred RNAs, our data reveal a much more complex organization. Surprisingly, none of the mRNAs or lncRNAs showed exclusive localization to a region in the egg, and none showed even localization along the A-V axis. This observation is in line with the traditional dogma of developmental biology that cellular determination during early development is caused by gradients of biomolecules along the developmental axis rather than by a biomolecule being exclusively present or absent in a particular region, which, as shown here, applies also for the egg itself. In *Xenopus laevis*, majority of RNAs, referred to as animal, are localized in the first third of the egg measured from the animal pole. We have previously argued that the animal intracellular profile forms spontaneously through diffusion^18^. Hence, the majority of maternal RNAs remains in and around the germinal vesicle/nucleus where they were produced. Colocation of animal mRNAs and the nuclear region is here further supported by profiling data at later stages of development, where the concentration of animal mRNAs is proportional to the number of nuclei in the blastula. Around 230 mRNAs form vegetal and extremely vegetal profiles^25,26,28,31^. Here, we discover a new localization category for mRNAs referred to as extremely animal. More than 400 mRNAs showed extreme polarization towards the animal pole and the animal pole cortex for subsequent distribution to animal blastomeres. The extremely animal category includes RNAs coding for transcription factors behind ectodermal tissue differentiation, such as Foxi2^32,33^, TGF-beta signaling inhibitor, and ectoderm determinant mRNAs including Dand5 (also known as Coco)^34,35^. *slc18a2* and *lima1* are also among the extremely animal mRNAs and are later found in tissues derived from ectodermal cells^26^. Our finding is plausible considering that ectoderm is derived from the animal part of the egg. Our discovery of an extremely animal polarized RNA fraction suggests there is a yet undiscovered transportation mechanism active during oogenesis that generates this profile. Several laboratories have in recent years, published large-scale spatial expression analysis of *Xenopus* early development using egg pole segments^26,31^ and single cells from 8-cell stage^21^. Their findings of asymmetrically localized RNAs along the A-V axis overlap with our observations.

Localization mechanisms have been described for the vegetal and extremely vegetal mRNAs^8^. They involve RNA-binding proteins and transportation complexes with specific localization motifs, “zipcodes”, presumably located in the mRNAs 3′UTR. Identification of the vegetal localization motifs and understanding their mechanisms have been subject to intense research including functional experiments, but only a few motifs have so far been identified and the mechanisms behind them remain elusive. We performed extensive sequence analysis to identify novel localization motifs specific for the vegetal and extremely vegetal mRNAs. Using several advanced sequence analyses tools we have identified putative vegetal localization motifs, some of which contain previously described CAC-rich sequences^14^. Majority of vegetal motifs were overrepresented in the 3′UTRs of vegetal and extremely vegetal mRNAs compared to the animal mRNAs and even more when compared to the extremely animal mRNAs. This observation indicates their relevance for vegetal RNA localization. Our analysis of the 3′UTRs of the extremely animal mRNAs identified no enriched motifs, suggesting they may be in the 5′UTRs or in the coding regions or perhaps even in trans sequences. Extensive kmer analysis of 3′UTRs of extremely animal and vegetal + extremely vegetal RNAs revealed no differences for sequences containing 3, 6, and 7 bases, but several sequences containing 4 and 5 bases were overrepresented and even unique in the vegetal sequences. Majority of those sequences showed good overlap with motifs revealed by DREME, MEME, and GIBBS. This observation supports the idea that localization motifs for the vegetal and extremely vegetal mRNAs are in the 3′UTRs. The length dependence observed suggests the localization motifs are 4–5 bases long.

The localization elements in the 3′UTRs that we found contain the previously known CAC-rich motifs and our newly discovered localization motifs (listed in Results). The motifs are statistically significant suggesting they are important. However, we do not as yet know their function. Our data set is too small to assess the importance of motif length, motif sequence conservation, order, and frequency of the different motifs, and many other factors that may contribute to the localization signal such as secondary structure elements. Several putative hairpin structures were also found during motif analysis that are enriched in the vegetal, extremely vegetal, and extremely animal categories (data not shown), and could perhaps be of importance.

Another known mechanism behind asymmetrical cell division is spatially regulated mRNA translation and mRNA degradation. Analysis of translation and stability regulatory sequences in *Xenopus* 3′UTRs revealed nearly universal presence, with few exceptions. However, limited database sources and depth of analysis make any prediction unreliable. Small noncoding RNAs such as miRNAs could also act as guides for specific mRNA degradation and they may silence translation. The activity of those mechanisms requires hybridization and depends on homology to form mRNA-miRNA duplexes. We performed miRNA localization analysis and found certain matches with mRNAs having the same localization, which suggests some miRNAs may be involved in spatial regulation of mRNAs during early development^18^. However, miRNA analysis remains challenging in *Xenopus*, because of the still incomplete annotation of noncoding RNA sequences and poor knowledge about the complex network of miRNA binding targets.

RNA profiles along the A-V axis have been known for many years. Here, we complement the earlier data with global profiling of lncRNAs and proteins. Global proteome mapping of oocytes and early-stage embryos is challenging because ~90% of the protein by weight is yolk. Our sample preparation has been optimized to decrease the yolk protein concentration, followed by extensive peptide prefractionation prior to MS. Nevertheless, low abundant proteins are challenging to detect, and many transcription factors important for the determination of the developmental plan may have escaped detection. Among the proteins detected, we find some with distinct preferential localization to either the animal or vegetal hemispheres, but for most proteins localization is unclear. For more than 70% of the egg proteins for which we conclude localization and know the localization of its mRNA, we find correlation: animal mRNA and animal protein, vegetal mRNA and vegetal protein, animal mRNA and even distribution of proteins. The latter is considered correlation, as the differences between protein profiles are not as dramatic as those for mRNAs, and many of the proteins found evenly distributed were probably produced from mRNAs with the spontaneous animal polarization arising by diffusion from the nuclei. The correlation between mRNA and protein localization in the egg suggests that mRNA is synthesized in the nuclei, transported, and then translated. In this scenario, the mechanisms leading to localization act on mRNA rather than protein. A similar mechanism has been described in the oocyte and early embryo of *Drosophila*^36,37^. For the remaining proteins, localization does not match that of their mRNA, but only two proteins show opposite profiles to those of their transcripts: *mcts*/Mcts1 (protein vegetal and mRNA extremely animal) and *trim36*/Trim36 (protein animal and mRNAs extremely vegetal). We are unsure as to the underlying mechanism that is contributing to these contrasting profiles for those unique cases. Differences in RNA/protein localization could be caused by active transportation of protein or RNA molecules after translation, alternatively RNA molecules could be degraded after translation to control the level of a particular protein in the specific region or protein stability/function are controlled by external factors causing molecule asymmetry.

The correlation between protein and mRNA localization we observe for the egg is not maintained throughout development. Sun *et al*., measured expression for nearly 4,000 proteins at eight stages of development and found significant discordances between transcript and protein expression levels that varied across stages^7,38^.

**Figure 8:**
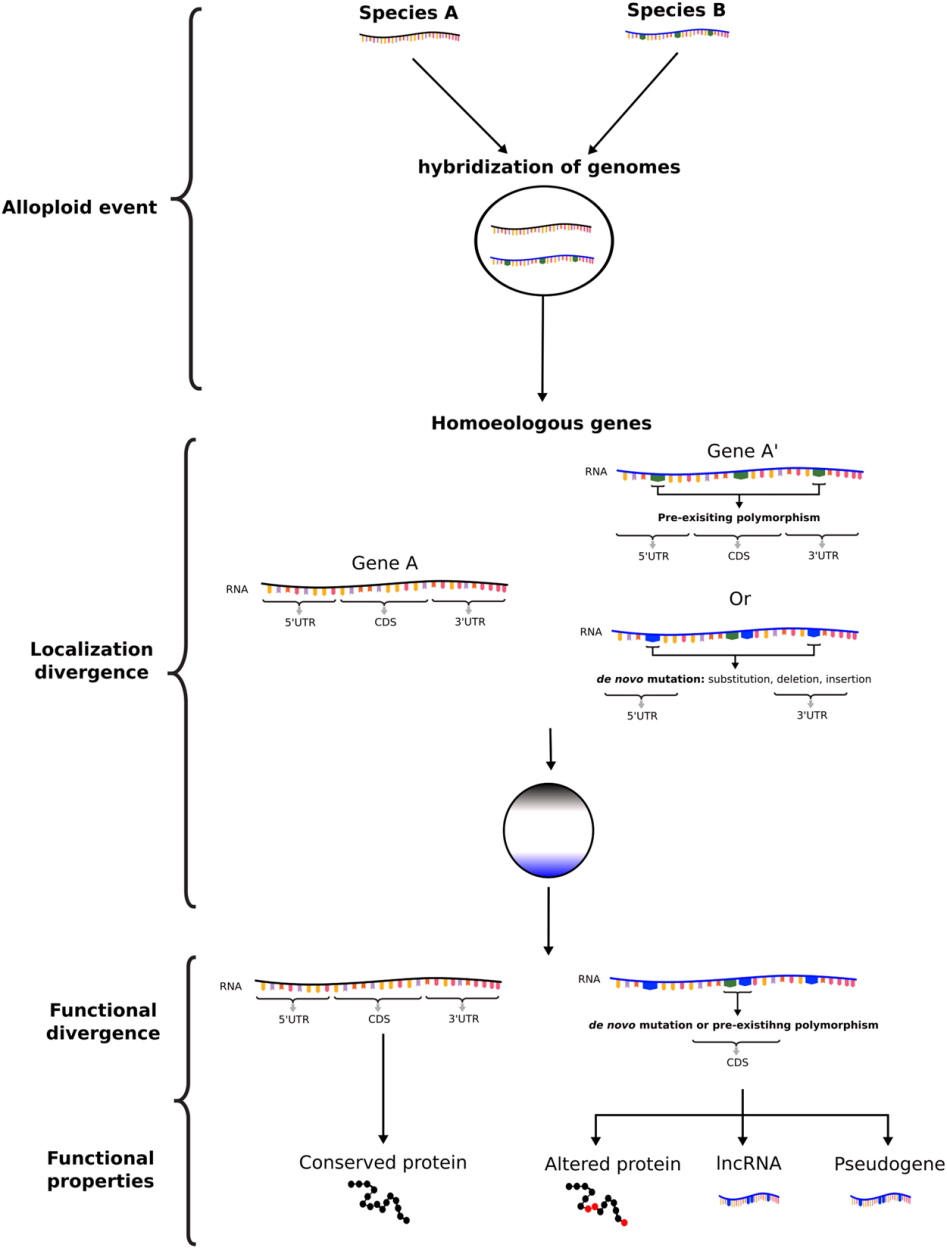
Scheme proposing altered gene function development based on species hybridization followed by asymmetric localization and modification of coding sequence.

It was observed that most homoeologous transcripts localize the same way within the *X. laevis* egg. However, a few homoeologues were observed with diametrically contrasting profiles, for example the L and S forms of the *exd2* gene. Orthologues of this gene have been previously described as a vegetally localized germ-plasm determinant and its product has been suggested to play a role in germ cell formation in both *Xenopus* and *Drosophila*, and mitochondrial translation in *Drosophila*^25,39^. Our results show that the transcripts of the *exd2.S* homoeologue are localized vegetally, while the *exd2.L* transcripts are located animally (Fig. 6D). Inspecting the genomic sequence data, we found that Exd2.S has lost a string of 30 amino acids, suggesting that the L and S versions may have diverged functions. Such sequence divergence is not uncommon for homoeologous or paralogous genes and have been previously documented^40,41^. Additionally, in yeast it has also been observed that duplicate genes may diverge to have different subcellular localization^41^. Research by Session, A. M and his colleagues found that the S chromosomes appear to have accumulated more mutations versus the L form, which consequentially resembles more the ancestral parental genome^30^. It is possible from our observation for the few genes that show contrasting localization profiles, that one form, perhaps the L form, has remained stable over time while the other, perhaps S homoeologue, has accumulated sufficient mutations to alter its subcellular location and possibly even its function (Fig. 8). Unfortunately, our quantitative protein data cannot distinguish between the two versions of Exd2, as only specific peptides coded by *exd2.L* were detected in addition to several peptides with shared sequences for Exd2.S and Exd2.L and we were unable to test this hypothesis on the protein level.

## Materials and Methods

### Sample preparation for RNA-Seq

*Xenopus laevis* females were stimulated with 500 U of human gonadotropin and eggs were collected the following day. Jelly coat was removed by five minutes treatment with 2% cysteine. 20 eggs were embedded into a single block of optimal cutting temperature (OCT) medium. Using forceps, the eggs were oriented with the animal cap towards the top and then frozen using dry ice. The OCT blocks were incubated for 15 minutes in a cryostat chamber before fastening to the holder and processed into 30-μm sections. Sections were immediately collected into precooled tubes with seven consecutive sections per tube. On average, 35 sections were prepared from each egg block. Three blocks each containing 20 eggs were prepared from different females to serve as biological replicates. All experiments were approved by the animal committee of the Czech Academy of Sciences and were performed according to EU legislation (including animal handling guidelines and regulations).

Total RNA was isolated from each pool of seven sections using 500 µL of Trizol (Sigma-Aldrich) according to the manufacturer’s protocol, followed by LiCl precipitation to remove inhibiting substances. Absence of inhibitors was verified using an RNA spike (TATAA Biocenter). Total RNA concentration was determined with a NanoDrop 2000 (ThermoScientific) and the quality of the RNA was assessed using the Experion system (Bio-Rad, Fig. 1A). Ribosomal RNA was depleted with the GeneRead rRNA Depletion Kit (Qiagen) and libraries were prepared using SuperScript II Reverse Transcriptase (Life Technologies) and TruSeq RNA Sample Preparation v.2 kit (Illumina). Library quality was assessed using the Fragment analyzer (Advanced Analytical) and quantified by absorption (NanoDrop ND-3300). Library pooling and sequencing were performed at BGI (Shenzen, China) using HiSeq 2500 (Illumina), 50bp pair-end.

### RNA-Seq data processing

Ribosomal RNA sequences were removed using sortmerna (version 2^42^) and the SILVA rRNA database (version 119^43^). Low quality reads and adaptor sequences were removed using Trimmomatic PE^44^. Reads were aligned against the reference *Xenopus laevis* genome version 9.1 (Xla.v91.repeatMasked.fa, Xenbase) using STAR (version 2.4.2a^45^). Annotated reads were counted using htseq-count^46^ with the reference model XL9.1_annot_v1.8.1.primary.gff3 (Xenbase).

Differential expression profiles were analyzed with DESeq^47^ and DESeq2^48^. Standard normalization could not be used because the main assumption of RNA-Seq normalization method is the equal distribution of the majority of genes between samples. But, this is not valid for intracellular localization, so, we introduced a new normalization strategy based on RT-qPCR quantification of representative candidates (Supplement Table 7). Normalization to the whole egg content was used for RNA distribution profile determination and details are described in our previous paper^17^. Measured RNA-Seq profiles were divided into five groups: extremely animal, animal, vegetal, extremely vegetal, and ubiquitous based on previous findings^17^. RT-qPCR assays were designed for *vegt-a* and *vegt-b* (vegetal)*; actb* and *akt1* (animal); *nanos1* and *rras2 (*extremely vegetal); *ifrd2, lima1*, and *slc13a4* (extremely animal); and *clic5* (ubiquitous). Three eggs from a different female frog were cryo-sectioned and analyzed using RT-qPCR tomography (method details published in^28^). qPCR Cq values were converted to size factors and used to normalize RNA-Seq data (SFig. 1B–D, details in supplemental Excel file). Finally, results after normalization and distribution analysis using DESeq and DESeq2 were compared. Genes with an average normalized read below 15 were considered too low expressed to be quantified reliably and were removed from analysis.

### Long noncoding RNAs (LncRNA) Identification

Bam files of aligned reads were merged using SAMtools (v. 0.1.19^49,50^) and then used for the generation of genome-guided *de novo* transcriptome. The *de novo* transcriptome assembled using Trinity (v2.3.2^51^, parameters –genome_guided_bam merged.bam –max_memory 120G –genome_guided_max_intron 50000 –CPU 12) was blasted against known annotated *Xenopus laevis* sequences (XL_9.1_v.1.8.3.2, blastn -query Trinity-GG.fasta -db XL_9.1_v.1.8.3.2_transcripts.fasta -out blastn.outfmt6 -evalue 1e-20 -max_target_seqs 1 -outfmt 6). Sequences that did not show significant hits or matched sequences with annotations starting with either “LOC”, “Xelaev” or “Xetrov”, were then blasted (using same criteria as above) against known long noncoding RNAs from *Xenopus tropicalis*^52^. Potential maternal lncRNAs were identified and their localization profiles were analyzed using the same parameters as for the localization analysis of mRNAs.

### RNA-Seq Data analysis

Eggs were sectioned and pooled into five segments designated A to E from the animal to the vegetal pole. Based on our previous results^17,18,28^ the following criteria were used to categories the intracellular profiles of mRNAs and lncRNAs: RNA-Seq data are available as GEO accession numbers (GSE104848).

Version 9.1 reference sequence for *Xenopus laevis* contains information about single nucleotide polymorphisms (SNPs) of homoeologous variants of the genes. The high quality and deep sequence coverage of our RNA-Seq data made it possible to distinguish RNAs produced from the long (L) and the short (S) chromosomes for homoeologous regions. Gene ontology was analyzed with WebGestalt^53^ using human gene symbols and genome reference.

### RT-qPCR

Ten nanograms of isolated total RNA was reverse transcribed into cDNA using SuperScript^TM^ III Reverse transcriptase kit (Invitrogen). The RNA was mixed with 0.5 µL of oligo-dT and random hexamers (mixture 1:1, 50 µM each), 0.5 µL of dNTPs (10 mM each), 0.5 µL of spike (TATAA Universal RNA Spike, TATAA Biocenter) and DNase/RNase free water to a total volume of 6.5 µL. The spike was included to test for unspecific bias in the quantification. The mixture was incubated for 5 min at 75 °C, 20 s at 25 °C followed by cooling to 4 °C for 1 min. 100 units of SuperScript III enzyme, 20 U of RNaseOUT (Invitrogen), 0.5 µL of 0.1 M DTT, and 2 µL of 5x First strand synthesis buffer were added to a final volume of 10 µL. The mixture was then incubated at 25 °C for 5 min, 50 °C for 60 min, 55 °C for 15 min, and 75 °C for 15 min. 50 microliters of water were added to the cDNA and the samples were stored at −20 °C. qPCR assays were designed using NCBI Primer-Blast (http://www.ncbi.nlm.nih.gov/tools/primer-blast/). Amplicon length was set to 90–200 bp and Tm to 60 °C. Specificity of all assays was confirmed by melting curve analysis measured from 65 °C to 95 °C in 0.5 °C intervals. qPCR mix contained 5 µL of iQ™ SYBR® Green Supermix (Bio-Rad), 0.5 µL of forward and reverse primers mix (mixture 1:1, 10 µM each), 2 µL of cDNA and water to reach a final volume of 10 µL. qPCR was performed on a CFX384 cycler system from Bio-Rad. PCR conditions were: initial denaturation at 95 °C for 2 min, 40 repeats of denaturation at 95 °C for 15 sec., annealing at 60 °C for 20 sec and elongation at 72 °C for 20 sec.

### RNA-Seq data verification

qPCR tomography was used to validate the RNA-Seq data. We selected five coding homoeologous genes (minimal difference in coding sequences), which, in contrast to the majority of the homoeologues, showed different localization profiles (*ctsc*, *naga*, *ctdspl*, *zfyve28.1* and *exd2*). Two genes (*dand5-like.L* and *slc18a2*) that showed extremely animal localization (see below) were also included in the validation. Primers were designed targeting sequences rich in SNPs (SFig. 2B) to distinguish between the L and S form of the transcripts. Three oocytes from different females were cryo-sectioned as described above and processed with qPCR tomography (^21^, SFig. 2C–H). Another verification of our results can be found in the recent large-scale studies using whole mount *in situ* hybridization^26,31^ to determine localization along A-V axis of selected animally and vegetally enriched RNAs.

### Localization motif search and analysis

Multiple EM for Motif Elicitation (MEME) (ver. 4.11.2^54^, Discriminative Regular Expression Motif Elicitation (DREME) (ver. 4.11.2,^55^) and GIBBS Motif Sampler (ver. 3.10.001,^56^) were used to identify putative conserved motifs. The three tools use different pattern recognition algorithms to find motifs. MEME uses probabilistic, positional constrained and discriminative motif discovery, while DREME performs discrete motif discovery, and the GIBBS sampler probabilistic motif discovery^57^. MEME and GIBBS sampler are based on Position Weight Matrices (PWM), with MEME using a modified version of the expectation maximization (EM) algorithm^54,56^. DREME can find shorter nucleotide motifs with higher confidence than MEME^55^.

3′UTR sequences of mRNAs from each localization group were analyzed for conserved motifs. Motif discovery using MEME was performed with and without the use of a position-specific prior (psp). Each position-specific prior was generated using psp-gen from the MEME-Suite and utilized genes originating from the other localization categories to serve as control set^57^. MEME analysis with psp, searched motifs assuming that each gene may contain either none or at most one copy of the motif, while MEME analysis without psp assumed that the genes could harbor multiple copies of the motif. A maximum of five motifs (or three motifs in runs without the psp) were requested during the analysis of the sequences from each localization category, with each motif being between six and 25 nucleotides.

DREME is inherently limited to detect short (≤8 nucleotides) motifs. Genes from the other localization groups were used as controls, and the significance of the found motifs was estimated with the Fisher’s Exact Test. Motifs with e-values (the enrichment p-value times the number of candidate motifs tested) above 0.05 were excluded.

GIBBS Sampler was run using the Eukaryotic default parameters, which include a recursive sampling mode, searching for five different motif patterns, with a maximum of five sites per sequence. Searched motifs had expected widths of 10, 10, 8, 10, and 10 bases and were expected to occur 18, 12, 13, 10, and 10 times due to chance. Number of seeds was set to 10, reverse complement to FALSE, background model to TRUE, fragmentation to TRUE, and MAP maximization to TRUE.

GIBBS analysis was performed on 3′UTRs from 44 extremely animal mRNAs and from 9 extremely vegetal mRNAs. DREME and MEME analyzed 3′UTRs of 149 vegetal, 28 extremely vegetal mRNAs, and 328 extremely animal mRNAs.

Motifs found enriched in the 3′UTRs of mRNA sequences from the vegetal, extremely vegetal, and extremely animal locations were selected for further analysis. The tool “Find Individual Motif Occurrences” (FIMO) (ver. 4.11.2) was used to scan the sequences within each group to confirm the presence of the identified motif^58^. Motifs with p-values lower than 0.001 were considered significant. From the FIMO data, the proportion of mRNAs with at least one copy of the motif was calculated for each localization category and the relative occurrence of the motif within each category was determined and presented as a heatmap.

Motifs that were identified after analyzing either the extremely vegetal or vegetal genes were categorized as vegetal motifs while motifs identified from extremely animal and animal genes were categorized as animal motifs. Similarity between the members within each motif group was determined using the motif aligning tool called STAMP^59^. Sequence comparison and alignment were done using Pearson’s correlation, Smith-Waterman Ungapped Alignment and Iterative Refinement. The alignment was used to construct a dendrogram based on UPGMA (Unweighted Pair Group Method with Arithmetic Mean) which was then used to sort and cluster the motifs within the heatmap. The dendrogram was used to manually group similar motifs into motif families. The selected similar motifs were then rerun through STAMP to produce a consensus familial binding profile.

### Kmer analysis

Significantly overrepresented and unique kmers within each localization category were identified and used to verify significance of the MEME, DREME, and GIBBS derived motifs. The frequencies of the kmers’ lengths, ranging from three to seven nucleotides, were derived from the 3′UTRs of vegetal (156), extremely vegetal^28^, and extremely animal (356) genes using the software R version 3.3.2 (2016-10-31) along with the Biostrings package version 2.42.1^60^. Upper tailed Chi-squared analysis using a p-value of 0.001 was used as criteria to consider a kmer as being overrepresented taking into account the length of the analyzed 3′-UTRs (Equation 1 using parameters: n = number of genes per localization category, O = observed number of kmer, E = expected number of kmer, N = length of 3′UTR; A = number of types of nucleotide {A, T, G, C}; t = number of repeats of kmer (i.e: 1); k = kmer length).

$$\begin{array}{rcl}{x}^{2} & = & \sum \frac{{(O-E)}^{2}}{(E)}\\ df & = & n-1\\ E & = & (N-t\ast (k-1),\,t)/{A}^{(t\ast k)}\end{array}$$

The significantly overrepresented kmers were then further filtered to identify those that were exclusive for each localization category. The MEME, DREME, and GIBBS derived motifs were then cross-referenced against each significant unique kmer.

In total, we analyzed 328 3′UTRs for the extremely animal RNAs that had a combined length of 270 kbp, three groups of 422, 427, and 417 3′UTRs for randomly selected animal RNAs with combined lengths of 776, 736, and 743 kbp, respectively, 149 3′UTRs for the vegetal RNAs with a combined length of 309 kbp, and 28 3′UTRs for the extremely vegetal RNAs with a combined length of 46 kbp.

### Proteome extraction and preparation

In one set of experiments, *Xenopus laevis* eggs and blastula embryos (stage 8) were individually embedded into a drop of water and frozen using dry ice. The egg was first divided into animal and vegetal hemispheres, which were then cut in the middle using a razor blade to obtain quarters. This way variations introduced by sectioning were minimized. In subsequent experiments, cryostat sectioning was used. Sections obtained from 20 eggs and embryos were pooled for analysis and three biological replicates of each egg and blastula stage embryos were prepared.

Bottom-up proteomic sample preparation approach coupling NP40 extraction with filter aided sample preparation (FASP) digestion has been well established in previous study^7^. Although NP40 extracts only ~30 % of the protein (~35 µg/embryo) compared to SDS, it excels in discriminating against yolk proteins, which resulted in more proteins being identified and quantified^61^. We followed this method in our experiments, but with minor modifications. Each collection of sections (A to D) was suspended in 100 µl of mammalian cell-PE LB^TM^ buffer (NP40) (G-Biosciences, St. Louis, MO) containing protease inhibitor (EASYpacks, Roche, Indianapolis, IN), homogenized for 60 s and sonicated for 5 min twice on ice. The lysates were centrifuged for 10 min at 12 000 g and the supernatant was collected in a fresh tube for protein concentration measurement with the bicinchoninic acid (BCA) method.

20 μg of protein from each section was reduced with 10 mM dithiothreitol (DTT, Sigma–Aldrich) for 1.5 hour at 37 °C and alkylated with 20 mM iodoacetamide (IAA, Sigma–Aldrich) in the dark at room temperature for 30 min^61^. The alkylated proteins were then transferred to a Microcon® −30 centrifugal filter unit (Merck, Darmstadt, Germany) with a membrane for sample cleanup and protein digestion based on the filter aided sample preparation (FASP) protocol^62^. Briefly, the lysis buffer was first removed by centrifugation at 18 000 g for 40 min. The proteins on the membrane were then washed three times with 200 µL of 8 M urea via centrifugation at 18 000 g for 40 min each time, and three times with 200 µL of 20 mM NH_4_HCO_3_ (pH 8.0, Sigma–Aldrich) via centrifugation at 15 000 g for 20 min to remove the urea. Finally, 50 µL of trypsin in 20 mM NH_4_HCO_3_ (pH 8.0) at an enzyme/substrate ratio (m/m) of 1:30 was added to the membrane and vortexed for 3 min followed by protein digestion at 37 °C for 16 hours.

The digests were collected by centrifugation. To improve recovery, the membranes were washed twice with 50 µL of 20 mM NH_4_HCO_3_ (pH 8.0). The digests were then acidified with formic acid (FA, Sigma–Aldrich), followed by peptide desalting with C18 spin columns (Pierce Biotechnology, Rockford, IL).

After lyophilization, peptides from each section were labeled with ‘isobaric tags for relative and absolute quantitation’ (iTRAQ) 8-plex reagents according to the manufacturer’s protocols (AB Sciex, Foster City, CA) with minor modifications. The lyophilized digests were dissolved in 12 µL of 500 mM triethylammonium bicarbonate buffer (pH 8.5). 55 μL of isopropanol was added to each iTRAQ reagent vial, and 24 µL of iTRAQ reagent was added to the digests. After labeling at room temperature for 2 hours, 50 µL of 100 mM Tris-HCl buffer (pH 8.0) was added to the samples and incubated at room temperature for 40 min to quench the reaction. The labeled samples in each experiment were then pooled as follows. Experiment I (E1): two replicates of sections A to D from the oocyte; experiment II (E2): two replicates of sections A to D from the blastula; experiment III (E3) the third replicate of sections A to D from both the oocyte and blastula. The three tubes with the labeled digests (E1, E2 and E3) were lyophilized and desalted with Sep-Pak C18 1 cc Vac Cartridge (Waters Corporation, Milford, MA).

### Strong cation exchange liquid chromatography fractionation

The labeled digests (E1, E2, and E3) were fractionated with strong cation exchange (SCX) liquid chromatography using a Waters Alliance HPLC system (Waters, Milford, MA). 100 µL of labeled samples in 0.1% FA was loaded onto a Zorbax 300-SCX column (2.1 mm i.d. × 150 mm length, 5 µm particles, Agilent Technologies). The mobile phase gradient was generated using buffer A (10 mM KH_2_PO_4_, 20% ACN, pH 2.8) and buffer B (1 M KCl in A, pH 2.8). After loading, the sample was washed for 20 min with 100% buffer A to remove excess iTRAQ reagent. The peptides were separated by a 60 min linear gradient from 100% A to 100% B. The column was then washed with 100% buffer B for 10 min and equilibrated with 100% buffer A. Flow rate was 0.3 mL/min. Eluates from 23 to 83 min were collected as 1 min per fraction (total 60 fractions) and each was vacuum concentrated. Fifteen samples were generated by pooling every 15th fraction (i.e., fractions 1, 16, 31, and 46 were pooled to generate sample 1, *etc*.) to obtain similar amount in each sample and desalted with C18 ZipTip® (Merck, Darmstadt, Germany).

### UPLC-ESI-MS/MS analysis

Peptides were analyzed using a nanoRPLC-ESI-MS/MS system, which consists of a nanoACQUITY UltraPerformance LC® (UPLC®) system (Waters, Milford, MA, USA) and a Q Exactive HF mass spectrometer (Thermo Fisher Scientific). Chromatography was performed using a commercial C18 reversed phase column (Waters, 100 µm  × 100 mm, 1.7 µm particle, BEH130 C18, column temperature 40 °C). The emitter (Silica TipTM, New Objective, Woburn, MA) with a 20 µm inner diameter and 10 µm tip was employed for nanospray. Solvent A (0.1% FA in water) and solvent B (0.1% FA in ACN) were used to establish a 105 min gradient, comprised of 14 min of 2%, then 1 min of 2–8%, 84 min of 8–28%, 1 min of 28–80%, and finally maintained at 80% of solvent B for 5 min, with a flow rate of 0.7 µL/min. The column was equilibrated for 10 min with 2% of solvent B at 0.7 µL/min before analysis of the next sample.

The Q Exactive HF mass spectrometer was operated in positive mode. The electrospray voltage was 1.8 kV, and the ion transfer tube temperature was 300 °C. A top-ten data dependent acquisition method was used. Full MS scans were acquired in the Orbitrap mass analyzer over m/z 350–1800 with resolution of 60,000 and the number of microscans set to one. The target value was 3 × 10^6^ and the maximum injection time was 50 ms. The dynamic exclusion duration was 30 s. Higher-energy-collisional-dissociation (HCD) was performed at normalized collision energy of 32% and the activation time was set as 0.1 ms. The resolution of the MS/MS scan was set at 30 000.

### Proteome data analysis

Raw files were analyzed using MaxQuant (ver. 1.5.3.30). MS/MS spectra were searched against the protein reference database from *Xenopus laevis* genome 9.1 downloaded from the Xenbase website. The parameters for the database search were: reporter ion MS2 (iTRAQ 8plex) was set as sample type, oxidation (M) Acetyl (K), Acetyl (protein N-term), and deamination (NQ) were set as variable modifications, carbamidomethyl (C) was set as a fixed modification, trypsin as the specific digestion enzyme. A maximum of two missed cleavages was allowed. MaxQuant started with an initial search with a precursor mass tolerance of 20 ppm followed by main search with a precursor mass tolerance of 4.5 ppm and fragment mass tolerance of 20 ppm. The false discovery rate (FDR) was set to 0.01 for both peptide and protein identifications. Proteins quantified by the same sets of peptides were grouped and reported as one protein group. The protein group table was filtered to remove proteins quantified from the reverse database. All compared samples were measured in one run to avoid inter-run bias. The protein intensities were then normalized to an average protein level in each sample to remove inter-sample variation. Global mean normalization of proteome data was selected as the most reliable approach for our samples. Proteome data are available at ftp://massive.ucsd.edu/MSV000081646.

Proteins were divided into three localization groups (when at least two of the three biological replicates showed the same profile):

Localization was classified as animal when the protein was most abundant in segment A and the difference in amounts between segments A and D > 5%.

Localization was classified as even when the protein amount in each segment A-D was between 20–30% and the relative standard deviation across segments A - D < 5%, or when the protein is most abundant in segment B and there is more in segment C than in D (reflecting the mRNA animal profile).

Localization was classified as vegetal when the protein was most abundant in either segment C or D and the difference between segment C or segment D and segment A was >5%.

## Conclusions

We show that mRNAs, long non-coding RNAs (lncRNAs), and proteins have distinct intracelular distributions in the *Xenopus laevis* egg, being more abundant either at the animal or the vegetal side or rather evenly distributed with a small polarization towards the animal side due to asymmetric localization of the nuclei. Polarization of mRNAs and proteins correlate, suggesting mRNA is produced in the nuclei, polarized by one of several mechanisms, and thereafter translated producing proteins. The extremely animal and vegetal polarized molecules retain their distribution throughout cell division at least to the blastula stage. Vegetal mRNAs are presumably polarized through mechanisms that interact with short sequence motifs of 4–5 bases. Some of these motifs are CAC rich, as reported before, but there are also other motifs. The mechanism behind the animal polarization of mRNAs remains unknown. Homoeologous variants of genes generally show the same intracellular profiles, validating our approach as internal controls. However there are rare exceptions, where homoeologous genes have acquired mutations that lead to opposite polarization within the egg and presumably different distribution across blastomeres during early stage development, and possibly having different biological functions. This behavior of homoeologous genes may be a novel mechanism of genetic evolution.

## Electronic supplementary material

Supplement dataset 1

Supplement dataset 2

Supplement dataset 3

Supplement dataset 4

Supplement dataset 5

Supplement dataset 6

Supplement dataset 7

Supplement Figures
