# Supplementary material for: Asymmetric distribution of biomolecules of maternal origin in the Xenopus laevis egg and their impact on the developmental plan
Source: Sci Rep. 2018 May 29;8:8315. doi: 10.1038/s41598-018-26592-1 (PMC5974320; doi:10.1038/s41598-018-26592-1)

**Asymmetric distribution of biomolecules of maternal origin in the *Xenopus laevis* egg and their impact on the developmental plan**

Radek Sindelka, Pavel Abaffy, Yanyan Qu, Silvie Tomankova, Monika Sidova, Ravinda Naraine, Michal Kolar, Elizabeth Peuchen, Liangliang Sun, Norman Dovichi, Mikael Kubista


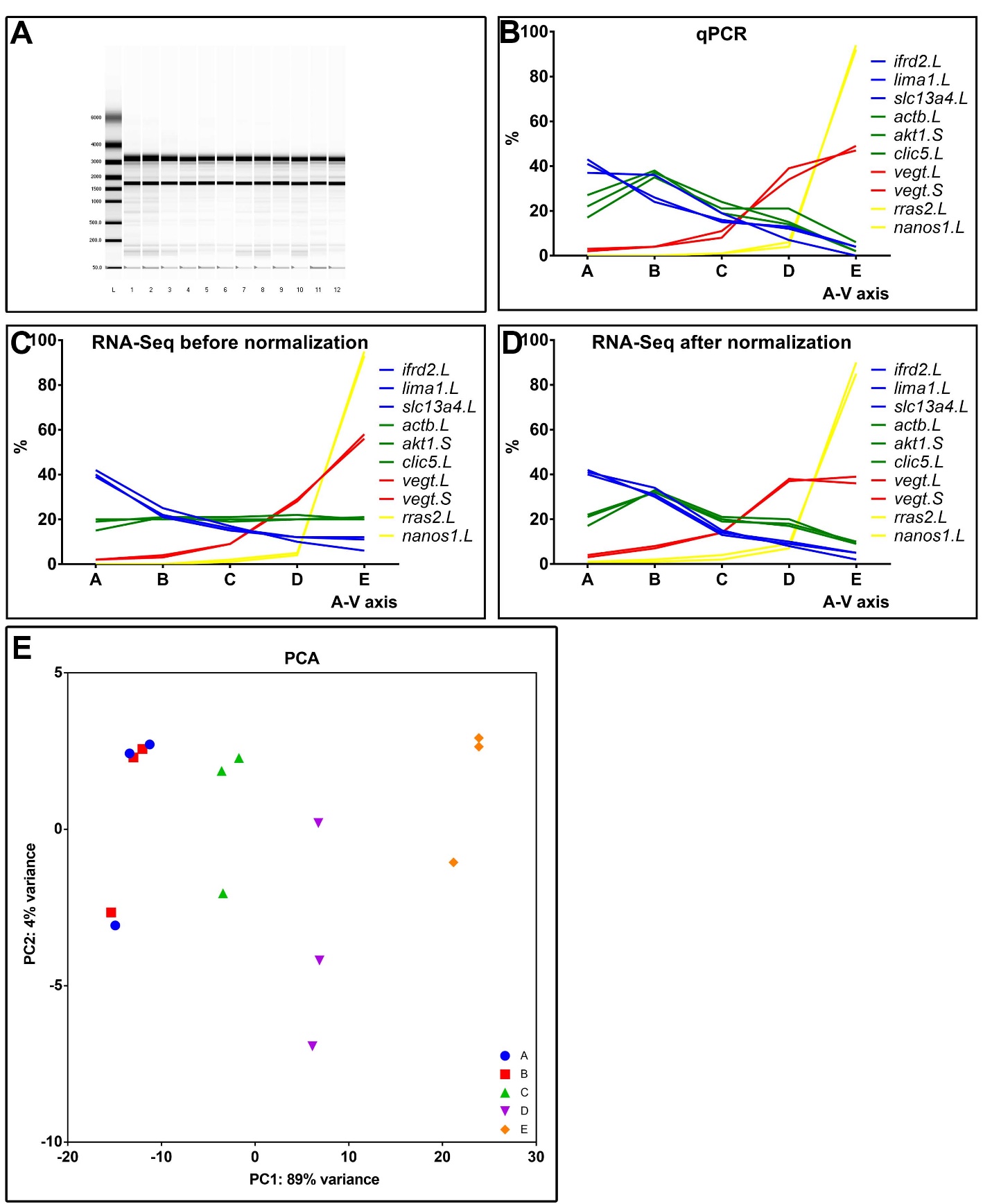


**SFig.1** Experimental quality control. Total RNA and library qualities were validated using Experion system (Bio-rad) (A). Normalization using qPCR tomography was used for RNA-Seq results (B-D). PCA analysis of animal-vegetal segments (labelled A, B, C, D, E) from three individual egg samples showed high correlation of RNA-Seq data.

**SFig.2** Validation of RNA-Seq data using qPCR| tomography. Correlation plot shows high similarity of localization profiles obtained using qPCR and RNA-Seq (A). qPCR primers were designed to distinguish homeologous forms of *naga*, *zfyve28* and *exd2* genes based on sequence polymorphism and assays for *ctsc* and *ctdspl* were designed in variable regions (B). Localization profiles determined using qPCR nicely correlate with RNA-Seq profiles for all candidates (C-H).


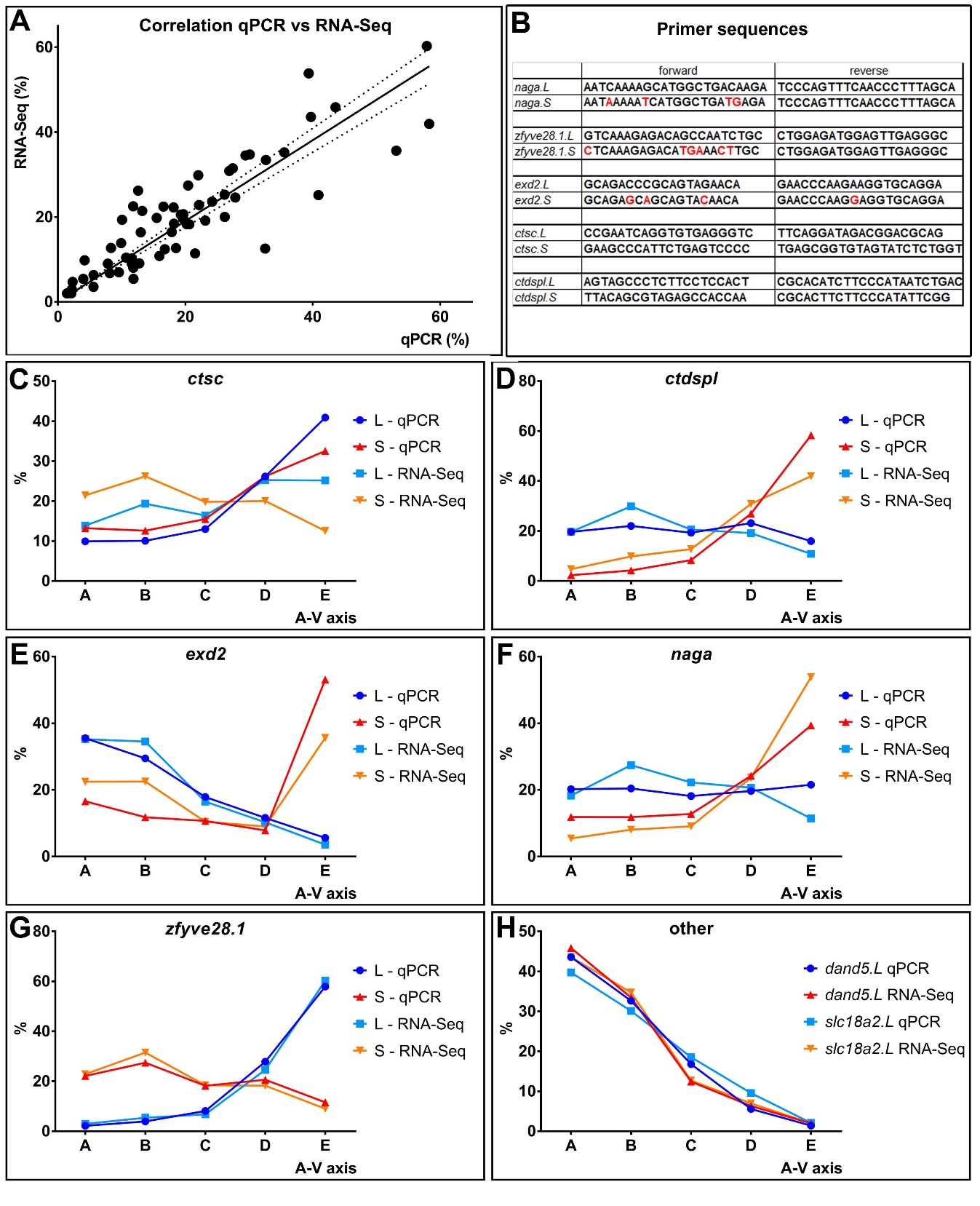


**SFig.3** Presence of vegetal motifs were analyzed in the 3’UTRs of three extremely vegetal (*ddx25*, *grip2* and *nanos1*) and three extremely animal (*dand5*, *ifrd2* and *lima1*) RNAs (A). Dependence of frequency on the motif length was tested using two vegetal motifs and no significant difference was observed (B).


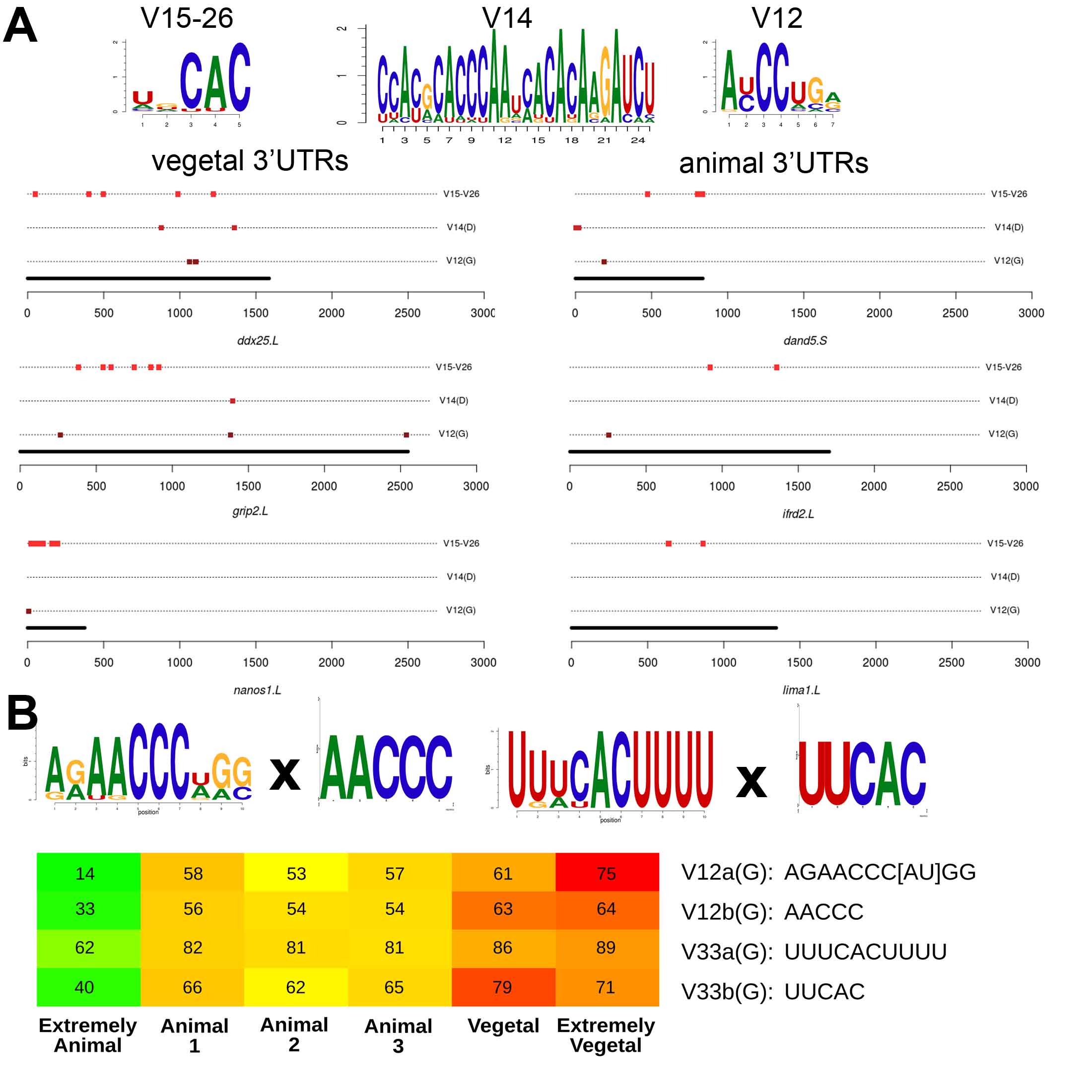

Supplement: Supplementary file 8 — Supplement Figures [file 41598_2018_26592_MOESM8_ESM.docx]
